# Development and content validation of two new patient-reported outcome measures for endometriosis: the Endometriosis Symptom Diary (ESD) and Endometriosis Impact Scale (EIS)

**DOI:** 10.1186/s41687-020-0177-3

**Published:** 2020-02-18

**Authors:** Adam Gater, Fiona Taylor, Christian Seitz, Christoph Gerlinger, Kamonthip Wichmann, Claudia Haberland

**Affiliations:** 1 Adelphi Values, Cheshire, UK; 2 Adelphi Values, Boston, USA; 3 Bayer AG, Berlin, Germany; 4 Obstetrics, Gynecology and Reproductive Medicine, University of Saarland, Homburg, Saar Germany

**Keywords:** Endometriosis, Patient-reported outcomes (PROs), Endometriosis associated pelvic pain (EAPP), Endometriosis symptom diary (ESD), Endometriosis impact scale (EIS), Health-related quality of life (HRQoL), Development, Qualitative, Content validity

## Abstract

**Background:**

Endometriosis is a common, chronic, impactful condition in women of reproductive age. In the absence of established sensitive and specific biomarkers, disease severity is determined by patient-reported symptoms and impacts. This article details the development of two new patient-reported outcome (PRO) measures designed to assess efficacy endpoints in clinical studies: The Endometriosis Symptom Diary (ESD) and the Endometriosis Impact Scale (EIS).

**Methods:**

The ESD and EIS were developed according to best practice and scientific standards (including the Food and Drug Administration (FDA) PRO Guidance) and with extensive input from women with surgically-confirmed endometriosis. Research included: a review of published qualitative literature; concept elicitation interviews in the US, Germany and France (*n* = 45) to explore the experiences of women with endometriosis and to inform ESD and EIS development; and cognitive interviews in the US and Germany (*n* = 31) to assess relevance and understanding of the ESD and EIS and usability of administration using an electronic handheld device. The FDA and the European Medicines Agency (EMA) as well as PRO and clinical experts were consulted throughout the process.

**Results:**

Pelvic pain was identified as the most frequent, severe and bothersome symptom for women with endometriosis. Pain was reported to be greatest during menstruation (dysmenorrhea) and during or after sexual intercourse (dyspareunia). Pain resulted in significant impairments in physical activities, work/study, social/leisure activities, household activities and sexual functioning. All women highlighted the emotional impact of endometriosis. Descriptions of pain and associated impacts were largely consistent across participants from the US and Europe, with the most notable differences being the words used to describe the location of pain (e.g., ‘pelvis’ vs. ‘abdomen’). Testing during cognitive interviews indicated that the ESD and EIS were well understood and consistently interpreted. Furthermore, all participants found the ePRO devices easy to use and no issues regarding visual presentation, selection of responses or navigation were identified.

**Conclusions:**

Evidence from extensive qualitative research supports the content validity of the ESD and EIS as patient-reported measures of the disease-defining symptoms of endometriosis and the associated impact on women’s lives. Future research will seek to establish the measurement properties of the measures.

## Background

Endometriosis is a common chronic condition estimated to affect as many as 10% of women of reproductive age [1]. The condition is characterised by chronic pelvic pain, dysmenorrhea and dyspareunia. Past research has indicated that women experience significant functional disability and deficits in health-related quality of life (HRQoL) [2–9] as a result of these symptoms. Accordingly, the costs associated with endometriosis in terms of direct healthcare expenditure and indirect costs (e.g., reduced work productivity) are considerable [10, 11].

While changes in the number and size of endometriotic lesions have traditionally been used to assess the efficacy of treatments for endometriosis [12–15], studies have suggested that the extent of lesions is only weakly associated with the severity of pain [16–18]. In the absence of established sensitive and specific biomarkers, the key symptoms and impacts associated with endometriosis can only be measured by direct reports from women themselves [19]. Therefore, there is a need for reliable and well-defined PRO measures that can be used to determine the clinical benefit of medical interventions.

Evidence of content validity (i.e., the extent to which the content of an instrument is an adequate reflection of the construct to be measured) [20] and is measuring what is important to patients within the intended context of use is often regarded as the most important measurement property of PRO measures [21]. To ensure content validity, concepts assessed by PRO measures should be informed by members of the target patient population and measures should be worded in such a way that is relevant, meaningful and consistently understood by this population. The Biberoglu and Behrman (B&B) scale has traditionally served as the standard clinical outcome assessment for endometriosis symptoms (including pelvic pain) in both clinical trials and clinical practice [22]. However, review of the B&B reveals a number of critical limitations (e.g., the B&B was developed primarily by clinicians with little to no direct involvement of women with endometriosis) that question the content validity of the measure and the extent to which it can be considered a reliable, valid and sensitive assessment of patients' experiences of endometriosis [23, 24].

To address these limitations, two new electronic PRO (ePRO) measures have been developed based on extensive involvement of women suffering from endometriosis in close accordance with the FDA PRO Guidance [21] and best practices established by the International Society for Pharmacoeconomics and Outcomes Research (ISPOR) PRO Good Research Practices Task Force [25, 26]. The Endometriosis Symptom Diary (ESD) is a patient-reported daily diary assessing the key symptoms of endometriosis, while the Endometriosis Impact Scale (EIS) assesses the impact of endometriosis symptoms over the past 7 days. Multinational qualitative research conducted to inform the initial development of the ESD and EIS and to provide evidence of the content validity of these measures is summarised.

## Methods

**Fig. 1:**
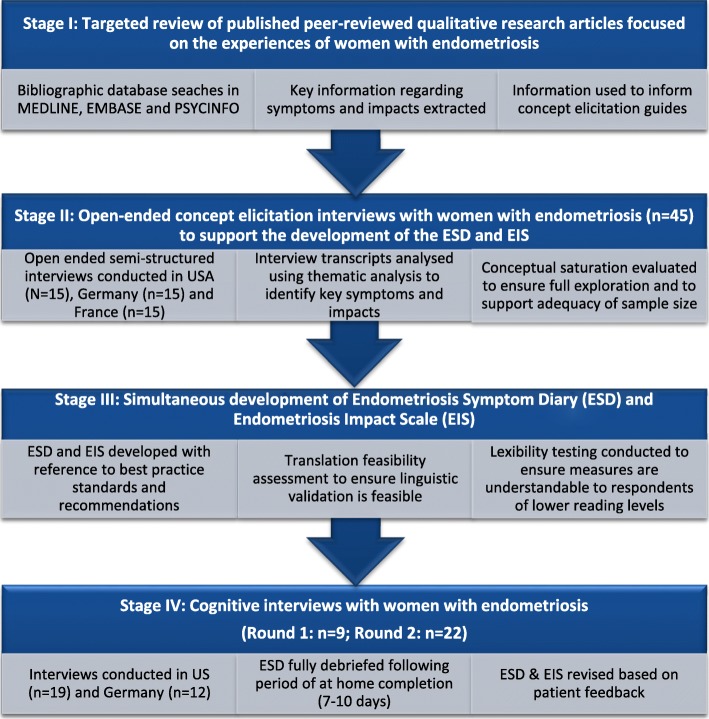
Overview of research stages to develop and evaluate the content validity of the Endometriosis Symptom Diary (ESD) and Endometriosis Impact Scale (EIS)

Development, refinement and confirmation of the validity of the ESD and EIS were conducted in stages, consistent with accepted best practice [21, 25, 26] (Fig. 1). The FDA and the European Medicines Agency (EMA) as well as PRO and clinical experts were consulted during the process.

### Stage I: targeted literature review

A targeted review of qualitative research studies in women with endometriosis was conducted to identify concepts that are relevant and important to women with endometriosis. Articles were identified via keyword searches conducted in MEDLINE, EMBASE and PSYCINFO. Searches comprised a combination of disease (e.g., ‘endometriosis’), data collection (e.g., ‘interviews’, ‘focus groups’) and analysis (e.g., ‘thematic analysis’, ‘grounded theory’, ‘discourse’, ‘phenomenological’) terms limited to adult participants and articles published in English in the past 10 years (2004–2014). Qualitative research articles exploring the symptoms and associated impacts of endometriosis were reviewed in full. Articles were excluded if qualitative methods or analysis were not used and if abstracts were not related to the experiences of women with endometriosis. Articles selected for full-text review were evaluated and salient information pertaining to study aim(s), sample demographic characteristics, methodology, and results were summarized. Key concepts relating to women’s experience of symptoms and impacts of endometriosis were used to inform the development of interview guides for subsequent concept elicitation interviews.

### Stage II: concept elicitation interviews

Semi-structured interviews were conducted with 45 women who had a surgically confirmed diagnosis of endometriosis to comprehensively understand the experience of endometriosis symptoms and the impact of these symptoms on various aspects of the womens' daily lives (e.g. physical activities, emotional well-being, sexual activities, paid work or study).

#### Recruitment

Women were recruited from the United States (US; *n* = 15), Germany (*n* = 15) and France (*n* = 15). A sample of 15 participants for each country was targeted with the aim of achieving conceptual saturation in each country. Conceptual saturation has previously been shown to be achievable in 12–15 individual interviews [27, 28]. EU countries with Latin-derived (i.e. France) and Germanic (i.e. Germany) languages were selected to promote linguistic and cultural diversity. Participants were recruited via referrals from treating physicians. Eligibility criteria for participation in the interviews were reflective of the criteria typically employed in clinical endometriosis studies. Specifically, all participants were required to have been diagnosed with stage I to IV endometriosis (according to revised American Society for Reproductive Medicine score classification) [29], as determined by laparoscopy or laparotomy in the past 5 years. Participants were also required to have recently experienced pain due to endometriosis – as verified by a participant-reported score of ≥3 on an 11-point numeric rating scale (NRS) assessing worst endometriosis-associated pain in the last 24 h at the time of screening (0 = no pain; 10 = pain as bad as you can imagine). Recruitment quotas were employed to ensure demographic and clinical diversity in the study sample.

#### Interview procedure

Interviews (lasting approximately 1 h) were all conducted face-to-face by female interviewers with extensive experience of conducting qualitative interviews among people with a variety of health conditions. Interviews were conducted in local language using a semi-structured interview guide (which was developed in US-English and formally translated for use in France and Germany). All interviewers received a detailed briefing on the study objectives, content of the interview guides and adverse event reporting procedures. All interviewers also engaged in a mock interview prior to the commencement of interviews.

Broad, open-ended questions were asked initially, with care taken not to lead or direct participant responses and to provide every opportunity for concepts to be mentioned ‘spontaneously’. Focused probes were only used to elicit feedback on potentially relevant concepts that did not arise spontaneously during the course of the interview. Prior to the interview, participants were asked to create a collage that ‘represented their experience of endometriosis’ which was subsequently discussed during the interview and used as a means to facilitate further spontaneous (i.e., patient-directed) elicitation of concepts.

#### Analysis

All interviews were digitally recorded, transcribed verbatim in the language in which they were conducted, and (for German and French transcripts) subsequently translated into US-English. A software package (Atlas.Ti) was used to facilitate the storage and qualitative analysis of interview transcripts using thematic analysis [30]. Each transcript was coded individually with the first two transcripts in each country used to create a coding scheme to be used throughout the analysis process by two separate analysts (the content and the data quality reviewer) to monitor and establish consensus in the coding scheme. As new codes emerged throughout the process, transcripts were reread and analysed to ensure all codes were consistently applied. All reported qualitative data were verified through a review of source data from the transcripts.

Saturation, defined as the point at which no new relevant or important information emerges with the collection of more data, was assessed to confirm that the concepts elicited by participants in each country had been fully explored [21, 28, 31]. Sequential sets of interviews (i.e.*,* interviews 1–5 vs. 6–10 vs. 11–15) were compared to one another. If no new concepts were elicited during the final set of interviews (i.e., interviews 11–15) then saturation was said to have been achieved.

### Stage III development of the ESD and the EIS

Based on information derived from the targeted literature review and concept elicitation interviews, draft items, instructions, response options and hypothesised conceptual frameworks for US-English, French and German versions of the ESD and EIS were developed. Input was sought from expert clinicians (to ensure the clinical relevance of items), linguistic validation specialists (to ensure the cross-cultural validity and translatability of the items) and ePRO vendors (to ensure ease of implementation of measures on electronic devices). Formal translatability and lexibility assessments were conducted to determine the appropriateness of the draft measures for adaptation to other languages and for use in respondents with low levels of literacy.

The ESD and EIS were developed for completion on a handheld electronic device. This has notable advantages over traditional pen-and-paper methods in terms of providing confidence as to when questionnaires have been completed (preventing back-filling or forward-filling of questionnaires), implementing safeguards for avoiding missed completions (e.g. through use of alarms) and minimising time and potential errors associated with subsequent manual entry of questionnaire data [32].

### Stage IV: cognitive interviews

Semi-structured cognitive interviews and pilot testing were conducted with women with endometriosis to evaluate the relevance and participant understanding of draft items, instructions and response options. The usability of the handheld ePRO device (TrialMax Touch eDiary; CRF Health, Plymouth Meeting, Pennsylvania, US) was also assessed during these interviews.

#### Recruitment

An independent sample (i.e., not including those women who participated in concept elicitation interviews) of 31 women with endometriosis in the US (*n* = 19) and Germany (*n* = 12) were recruited for participation in the cognitive interviews. Prior research has recommended cognitive interview sample sizes of 30 or more be preferred in order to achieve a reasonable power to detect prevalent problems [33]. Participants were subject to the same eligibility criteria employed during the concept elicitation interviews and recruitment quotas were implemented to ensure a diverse sample. Note that cognitive interviews were not performed in France due to difficulties identifying women eligible for participation during the prior concept elicitation interviews. Furthermore, a greater proportion of US participants were targeted for recruitment to account for demographic diversity in the US population.

#### Procedure

**Fig. 2:**
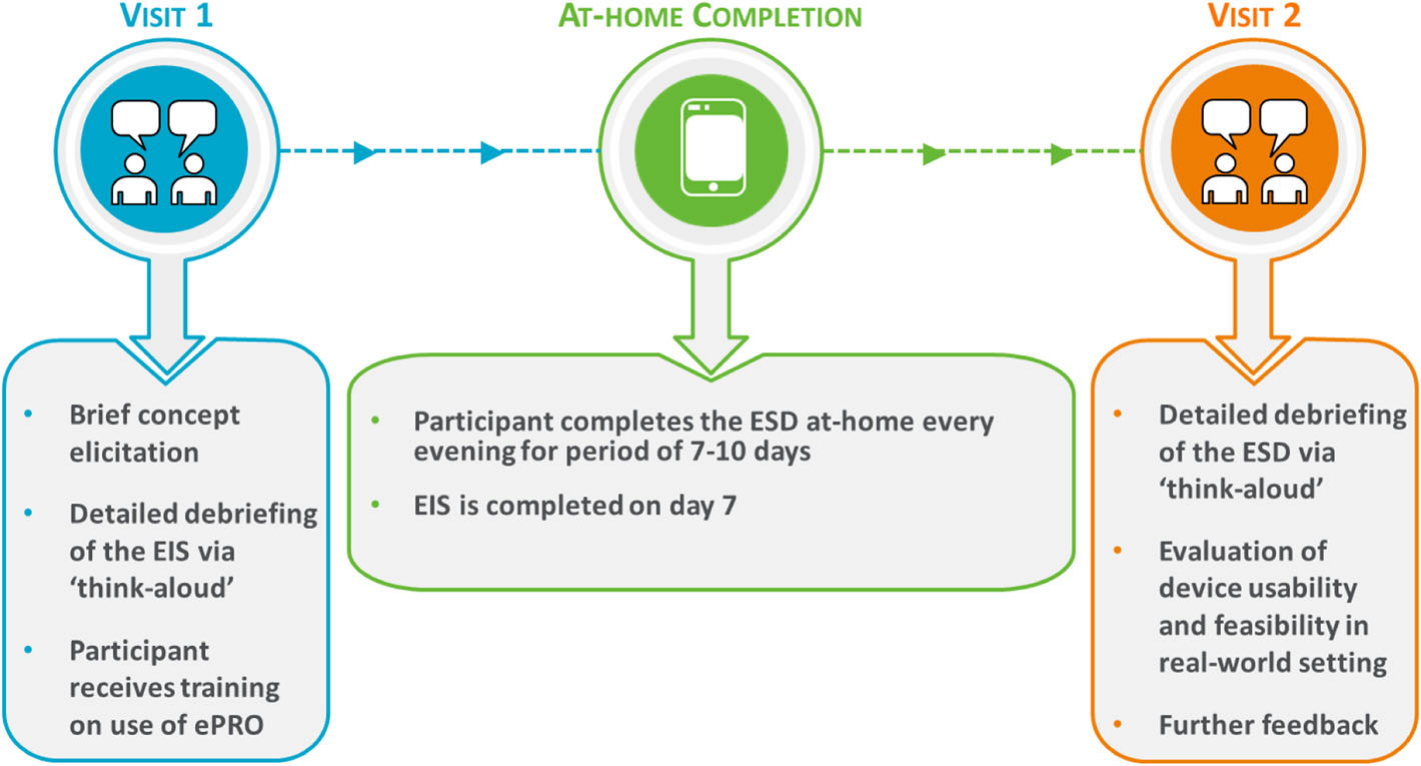
Overview of cognitive interview process

Each participant attended two study visits (Fig. 2). During visit 1, the participant’s understanding and comprehension of the EIS was assessed using a “think aloud” technique, whereby participants were asked to speak aloud their thoughts while responding to the EIS questions. Following the “think aloud” exercise, participants were asked about the relevance and understanding of EIS items, instructions and response options. The participants were also trained on use of the ePRO device, which they were required to take home for completion of the ESD on a daily basis for 7–10 days and completion of the EIS at the end of Day 7. This was designed to mimic how the questionnaires would be implemented in a clinical study. At visit 2, participants provided feedback regarding comprehension and understanding of the ESD using the “think aloud” method described above. Their experience of completing the ESD and EIS, including usability of the ePRO device was also explored. For both visits, a semi-structured interview guide was used to ensure that all areas of the ESD and EIS were discussed. Interviews were conducted in two separate rounds to allow implementation of modifications to the measures following round 1 (US, *n* = 5; Germany, *n* = 4), before testing in round 2 (US, *n* = 14; Germany, *n* = 8).

#### Analysis

Visit 1 and 2 interviews were digitally recorded and transcribed verbatim in the language in which they were conducted (with German transcripts subsequently translated into US-English). Atlas.Ti was used to facilitate analysis with participant quotes used to determine understanding/clarity and the relevance of each instruction, item and response option for each participant.

## Results

### Stage I: targeted literature review

A total of 14 articles met the pre-specified criteria for inclusion in the review [2–8, 23, 34–39]. Pain in the pelvic region was identified as the predominant symptom of endometriosis [2–8, 23, 35, 38, 39]. Pain was reported to be experienced at any time, although pain specifically associated with menstrual bleeding [7, 23, 38] and sexual intercourse [2, 3, 5, 23, 39] was commonly reported. Women with endometriosis characterized pain using a variety of sensory descriptors which can be broadly categorized as either continuous/constant or intermittent/short-term [7, 37].

Endometriosis-associated pain was reported to have a significant impact on numerous facets of women’s lives including physical functioning [7, 38, 39], ability to work [2, 6, 7, 38, 39] and to carry out activities of daily living (e.g., housework) [7], social functioning and personal relationships [2, 5–8, 38, 39]. Endometriosis was also reported to have a considerable emotional impact on women, with women feeling both depressed and irritable/moody [7]. In addition, dyspareunia was reported to significantly impact women’s sexual relationships; women frequently reported avoiding intercourse because of expected or experienced pain, and indicate that this puts strain on the relationship with their partner [2, 3, 5, 7]. Further impacts reported include impaired sleep, inability to concentrate and reduced appetite [7].

### Stage II: concept elicitation interviews

Table 1 shows the demographic and the clinical characteristics of women who participated in the concept elicitation interviews.

**Table 1:** Demographic and clinical characteristics of study samples used to establish the content validity of the ESD and EIS

Demographic and clinical characteristics	Concept Elicitation (*n* = 45)	Cognitive Debriefing (*n* = 31)
Age		
Mean (Range)	33.9 (18–44)	36.3 (21–45)
Country n (%)		
United States	15 (33.3)	19 (61.3)
Germany	15 (33.3)	12 (38.7)
France	15 (33.3)	N/A
Ethnic Background n (%)^a^		
Caucasian or White	10 (66.7)	8 (42.1)
African American	3 (20.0)	9 (47.4)
Other	2 (13.3)	2 (10.5)
Education Level n (%)		
High school diploma or GED	4 (8.9)	3 (9.7)
Some years of college	8 (17.8)	5 (16.1)
Certificate program	7 (15.6)	9 (29.0)
College or University degree	1 (2.2)	10 (32.3)
Graduate or professional degree	25 (55.6)	4 (12.9)
Experience of pain at screening n (%)^b^		
No pain (0)	0 (0.0)	1 (3.2)^d^
Mild (1–4)	16 (35.6)	9 (29.0)^d^
Moderate (5–6)	14 (31.1)	8 (25.8)
Severe (7–10)	15 (33.3)	13 (41.9)
Mean (range)	5.8 (3–10)	5.9 (0–10)
Current treatment n (%)^c^		
Painkillers	22 (48.9)	19 (61.3)
Oral contraceptives	13 (28.9)	11 (35.5)
Other hormonal contraceptives	3 (6.7)	5 (16.1)
GnRH analogue	4 (8.9)	5 (16.1)
Progestin	3 (6.7)	1 (3.2)
Other	3 (6.7)	1 (3.2)

^a^Data not available for European sample; therefore, percentages calculated based on US sample only (concept elicitation: *n* = 15, cognitive debriefing: *n* = 19)

^b^Self-rated assessment of endometriosis-associated pain at its worst in the last 24 h (using a 0–10 NRS where 0 = no pain and 10 = pain as bad as you can imagine). Classifications of pain based on Serlin et al. (1995) [40]

^c^Counts not mutually exclusive

^d^Of note: Two patients from the US sample had NRS scores < 3

Pain was mentioned by all 45 participants (96% of who mentioned this spontaneously) and was described by the vast majority of participants as being the most frequent (92%), most severe (92%) and most bothersome (86%) symptom that they experience:

- *“The pain, that’s for sure. The nausea, the fatigue, and the dizziness and all, I’m sure I could deal with a lot better if they were alone”* (101)^1^
- *“If the pain could be wiped out, then nothing else has really been troublesome. It’s just the pain.”* (104)

Participants used a variety of sensory descriptors to describe their experiences of pain, such as sharp/shooting/stabbing (*n* = 32), cramping/contractions (*n* = 26), and dull/aching pain (*n* = 16). Two distinct types of pain were identified (‘constant’ and ‘short-term’ pain). The sensory descriptors used to describe these types of pain were not mutually exclusive, rather these types of pain were instead differentiated by temporal characteristics.

While pain was typically considered to be at its greatest during menstruation, participants reported experiencing pain throughout the entire menstrual cycle (including pre-menstrual pain, menstrual pain, post-menstrual pain and non-menstrual pain). Participants indicated that pain occurring outside a menstrual period frequently could not be differentiated from cyclical pain, nor dissociated from pain with periods. Pain was also reported both during (*n* = 24) and following sexual intercourse (*n* = 16).

When asked about the location of their pain, participants most commonly referred to the pain occurring in the pelvic region (including uterus, ovaries, and bladder; *n* = 37), abdominal region (including stomach; *n* = 40), and lower back (*n* = 36). Pain in the legs was also mentioned by participants (*n* = 24); however, this was largely described as being a result of pain radiating down from the pelvic region. Pain descriptions were largely consistent across participants from the US and Europe; the most notable differences being the words used to describe pain location (e.g., ‘pelvis’ vs. ‘abdomen’).

In addition to pain, many women (*n* = 33) referred to vaginal bleeding, including heavy menstrual bleeding (*n* = 30) and unpredictable bleeding or spotting outside of their usual menstrual cycle (*n* = 14) (Table 2).

**Table 2:** Concept elicitation interviews: overview of key symptoms reported by women with endometriosis (*n* = 45)

Concept	Sub-concept	Example Quote (Patient ID#)
Pain (*n* = 45)	Constant pain (*n* = 40)	“*The consistent ache - you see, because the consistent ache, the dull ache is there every single - it’s hurting right now as I’m sitting here talking to you.”* (306)
	Short-term pain (*n* = 31)	*“I’ll get a pain in my pelvic area but it’s short-term. The duration isn’t like it is when my cycle is on.”* (104)
Dysmenorrhea	Pain during period (*n* = 43)	*“But on - during my menstrual cycle, when I was having my period, the pain was excruciating. At times it literally felt like a sword had been pierced right through my side, my left side”* (306)
		*“…and sometime the pain is just - you know, especially around my menstrual period, it’s very painful, that all I can do is lay on the couch and just - and I might take Tylenol, but sometimes that doesn’t even help.” (206)*
	Pre-menstrual pain (*n* = 28)	*“I have a lot of pelvic pain and it gets worse, I think, about the week before my period, it starts to get really bad. I get a lot of stomach pains, a lot of lower back pain and then I get the period and the bleeding. “(106)*
		*“Well, with my symptoms, they’re usually worse like the week before my period and during my period. So usually like the week before, I’m just in bed, very fatigued, feeling - just hurting, like cramping, like as if you would have menstrual cramps. I’m like that the week before my period, and then during my period, it’s like 100 times worse”.* (204)
Dyspareunia	Pain during intercourse (*n* = 24)	*“I’ve had intercourse a couple of times, and sometimes that’s been very painful… It’s like a sharp pain. And then it’s a sharp shooting pain, and then it’s throbbing together afterward.” (206)*
	Pain after intercourse (*n* = 16)	*“The same type of pain, it sometimes stayed for 24 h, sometimes a few hours, sometimes… sometimes 2 or 3 days and then… that’s it.” (501)*
Vaginal bleeding (*n* = 33)	Heavy bleeding (*n* = 30)	*“They’re extremely heavy, like bad heavy. It doesn’t lighten up until like the last day.” (*207)
	Spotting (*n* = 14)	*“I’ll actually sometimes, like if I push myself too far, I’ll start bleeding. And sometimes it may just be like spotty bleeding.”* (302)

A range of other symptoms were reported by women with endometriosis including: tiredness (*n* = 40); headaches (*n* = 29); abdominal bloating (*n* = 26); nausea (*n* = 24); constipation (*n* = 14); vomiting (*n* = 12); dizziness (*n* = 12); diarrhoea (*n* = 9); lack of energy (*n* = 9); loss of appetite (*n* = 8); bloody stools (*n* = 7); frequent need to urinate (*n* = 7); feeling of heaviness (*n* = 6); painful breasts (*n* = 6); weight loss/weight gain (*n* = 6); and fever (*n* = 5). These symptoms, however, were mentioned much less frequently than pain associated with endometriosis and bleeding irregularities, and in the majority of participants were not considered to be linked to their endometriosis. As such, these may be considered secondary rather than primary symptoms of endometriosis (something later confirmed via discussions with expert clinicians).

At its worst, endometriosis-related pain was extremely debilitating for women, impacting many facets of their lives: *“Because it’s painful. It hurts. I don’t like the way it feels. It disrupts my whole life at that time that it’s going on.”* (304). Participants spoke in general about how the pain affected their usual tasks and activities on a daily basis: *“See, the stabbing pains are bothersome because they affect all my daily activities. Not only what I do, but what I would want to do”* (306). Such impairments manifested in impaired ability to participate in: physical activities (*n* = 44), work and study (*n* = 39), social and leisure activities (*n* = 35) and household activities (*n* = 31) and sexual activity (*n* = 30/41). Participants also reported an impaired ability to sleep (*n* = 36), concentrate (*n* = 28) and eat (*n* = 8). Use of prescription or over the counter pain medications was common among patients (*n* = 39/45). Indeed, reports from patients implied that pain medication was an integral part of managing their condition and minimizing the impact of endometriosis on their daily lives: *“And then, I take analgesics… otherwise I can’t work, in fact.”* (508). All 45 women interviewed referred to the significant emotional impact of endometriosis (Table 3).

**Table 3:** Concept elicitation interviews: overview of key impacts reported by women with endometriosis (*n* = 45)

Concept	Sub-concept	Example Quote (Patient ID#)
Physical activities (*n* = 44)^a^	Lie down (*n* = 42)	*“It was intense pain in … at times I really felt too much pain in my abdomen, and I felt that the only way … for it to go was to lie down … and I had to lie down.”* (503)
	Walking (*n* = 22)	*“I can hardly walk, and as I said, without pain is out of - pain killers - is out of question.”* (406)
	Standing (*n* = 25)	*“And some days I was like I can’t even stand up because I’m in pain.”* (305)
	Sitting (*n* = 14)	*“I can’t drive for long periods of time. I can’t sit up straight for long periods of time.”* (306)
	Lifting (*n* = 16)	*“Yeah, that when I get the pain, I’m like I can’t pick kids up. It hurts. My stomach hurts.”* (301)
	Carrying (*n* = 16)	*“There are heavy things, let’s say, when my abdomen hurts too much, anything heavy I don’t carry it.”* (505)
	Exercise (*n* = 28)	*“Running, jogging, that’s absolutely out of the question. I used to run two miles a day, and weight train for an hour a day, and I can’t do that anymore”* (306)
Emotional state (*n* = 45)	Sad (*n* = 21)	*“Yes, the sadness because my kids want to do things, and we don’t go out and stuff sometimes.”* (207)
	Depressed (*n* = 22)	*“Because it makes you depressed, because you are in bed.”* (204)
	Irritable (*n* = 13)	*“Like it [the pain] just makes me irritable, like I said.”* (207)
	Bad mood (*n* = 23)	*“When I am in pain, I have mood swings and I know the pain causes them.”* (513)
	Angry (*n* = 15)	*“I just get - when the pain comes on* * I get angry”* (305)
	Annoyed (*n* = 19)	“it was frustrating and annoying, because I had to leave work constantly to go to doctor appointments all the time” (301)
	Anxious (*n* = 23)	*“Just depression and anxiety because of pain.”* (305)
	Stressed (*n* = 10)	*“I feel stressed, just waiting at some point of the day to be ambushed by an excessive amount of pain”* (306)
Sexual activities (*n* = 30)^b^	Avoidance (*n* = 23)	*“I final stopped (having sex) altogether* *- because it was just no longer tolerable.”* (410)
	Limited enjoyment (*n* = 12)	“A*t each penetration… when it’s painful. So,* * it’s quite unpleasant, it’s unpleasant for me”* (509)
Work and study (*n* = 39)		*“Because when - I couldn’t even go to work if I’m having severe pain, so I’d have to call in sick.”* (206)
		*“At work or after, I used to be able to do more with my time but now it feels like I’m slowing down”* (201)
Social and leisure activities (*n* = 36)		*“Well, when the pain was very severe I would have liked to curl up like a hedgehog somehow, that one - that I be left alone - I do not want to have to deal with anyone”* (403)
		*“…my partner and a bunch of friends were having a big potluck dinner at their house. And they live across town, and it would have been a bike ride and I wasn’t feeling well, so I didn’t go.”* (101)
Household activities (*n* = 27)		*“…when I’m not feeling good, then it just kind of sits there and then when I feel better, I go in and do it.”* (106)
		*“It slows me down like with all the usual things I have to do at home.”* (302)
Impaired sleep (*n* = 36)		*“A lot of times I’ll wake up in pain. If I’ve slept through my next opportunity to take another dose of pain medication, then the pain will wake me up and I’m just sort of awake after that.”* (101)
Impaired concentration (28/45)		*“I mean, trying to drive a car and you’re in pain. Well, that’s taking your - you’re distracted. You’re thinking about that, not what you should be. You’re not thinking about your job or everyday life activities.”* (301)
Eat less (8/45)		*“Because the pain’s so bad I feel sick to my stomach. I feel like I can’t eat.”* (301)

^a^ Number of participants reporting impact during CE interview

^b^Note that four study participants commented that they were not sexually active at the time of the study

Consideration of qualitative data obtained during the interviews revealed that no new concepts were elicited during the final set of interviews and that saturation was achieved within this sample. Ninety percent of all concepts were elicited in the first round of five interviews in each of the three countries (US, Germany and France). Furthermore, all concepts identified were elicited in each country.

### Stage III: development of the ESD and the EIS

#### ESD

The ESD was developed as a patient-reported electronic diary to assess the key symptoms associated with endometriosis: pelvic pain, dysmenorrhea and dyspareunia. The ESD is designed to be completed once daily, with a recall period of the past 24 h. This recall period was selected to account for the day-to-day variability in the presentation (i.e., menstrual pain and non-menstrual pain, event-driven dyspareunia) and severity of endometriosis symptoms and to minimize recall error associated with asking respondents to recall their experiences over a long period of time [21, 41]. The draft ESD comprised 12 items.

ESD pain items instruct respondents to rate their pain ‘at its worst’, as there is evidence suggesting that ratings of worst pain are more reliable than reports of average pain and are most representative of the burden of pain [24, 42]. ESD items assessing pain utilise a numeric rating scale (NRS) ranging from 0 (‘no pain’) to 10 (‘pain as bad as you can imagine’); a widely used measure of pain intensity [43–46] recommended for assessment of pain in clinical trials [47] and measurement and assessment of pain associated with endometriosis [24, 48]. Past research indicates that NRSs are sensitive to changes in levels of pain and are easily understood by respondents [49].

PRO measures historically implemented in endometriosis clinical trials (e.g., B&B) make specific reference to the location of pain experienced by women (for example: ‘pelvic pain’ or ‘abdominal pain’). It is important to differentiate pain that may realistically be associated with endometriosis from other types of pain that may be experienced by women with endometriosis (e.g., headaches, breast pain). However, as demonstrated by findings from the concept elicitation interviews, women with endometriosis frequently experience pain in more than one region and use a variety of different terms to describe the location of pain. Such differences in terminology were notably evident when comparing data between countries. For example, when provided with a diagram on which to circle their areas of pain, women in each country highlighted similar areas. However, when describing the pain location verbally, US participants more commonly used the term pelvic, while pain in the abdomen was the most prominent location descriptor used by participants in Germany and France. Therefore, to facilitate comprehension and understanding, the ESD and EIS include a diagram (depicting front and rear-view body maps) that highlights the areas in which women with endometriosis typically experience pain associated with their endometriosis. This area is referred as the ‘target area’ and referenced throughout ESD and EIS items and instructions in place of verbal descriptors of pain location. The use of the term ‘target area’ avoids the use of specific clinical terminology which may be difficult for low literacy respondents to interpret and may vary across languages and cultures.

Dysmenorrhea is widely recognised as a cardinal symptom of endometriosis and included in historical measures of endometriosis symptom severity (e.g. B&B). Feedback obtained during the concept elicitation interviews highlighted concerns regarding participants’ ability to reliably attribute their pain to bleeding or differentiate between menstrual pain and non-menstrual pain. Therefore, the initial draft of the ESD included items assessing ‘worst pain due to your period’ as well as a daily assessment of vaginal bleeding (‘none’, ‘spotting’, ‘light’, ‘normal’, ‘heavy’). The inclusion of a daily assessment of bleeding is consistent with recommendations from the Art of Science Endometriosis meeting [24] and prior feedback from the FDA and facilitates additional assessment of dysmenorrhea and non-menstrual pelvic pain based on independent assessments of pain (i.e., ESD item 1) and bleeding without relying on respondent attribution.

Items assessing dyspareunia were developed based on the findings from the literature review and qualitative interviews. It is important that any daily assessment only accounts for days where the respondent did engage in sexual activities and is therefore able to provide a rating of dyspareunia. For that reason, an initial item asking whether the respondent had (or did not have) sexual intercourse was developed.

Assessment of use of analgesics and pain-reliving medications is key for understanding womens’ experiences of pain and to help demonstrate treatment benefit in endometriosis [24] and such measures are included within generic pain assessments (e.g. Brief Pain Inventory-Short Form) and traditional assessments of endometriosis symptoms (e.g. B&B). Given the intended use of the ESD within the context of a clinical trial, the ESD includes items assessing use of both protocol-specified supportive pain medication as well as use of additional pain medication.

**Fig. 3:**
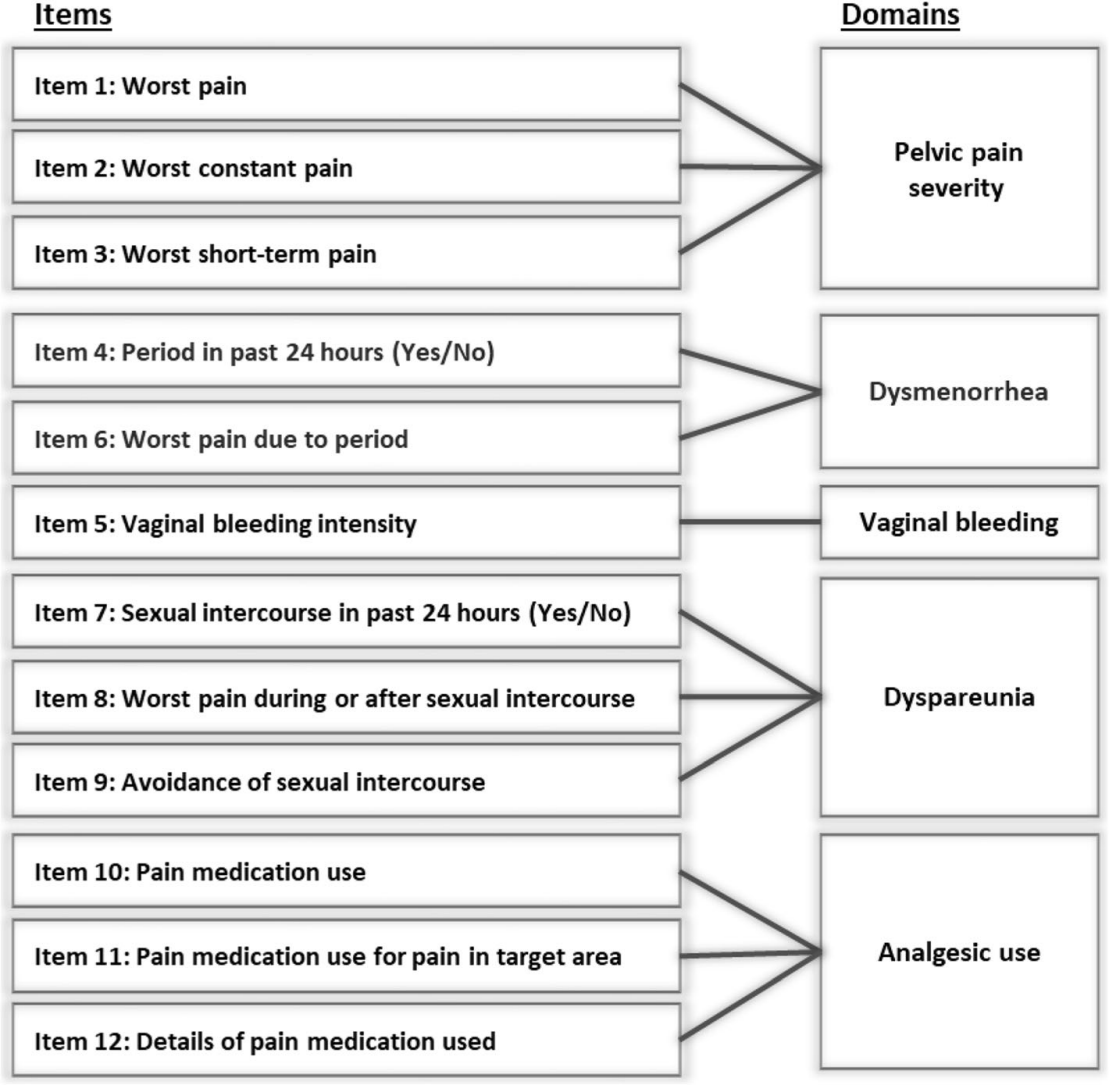
Endometriosis Symptom Diary (ESD) conceptual framework

**Fig. 4:**
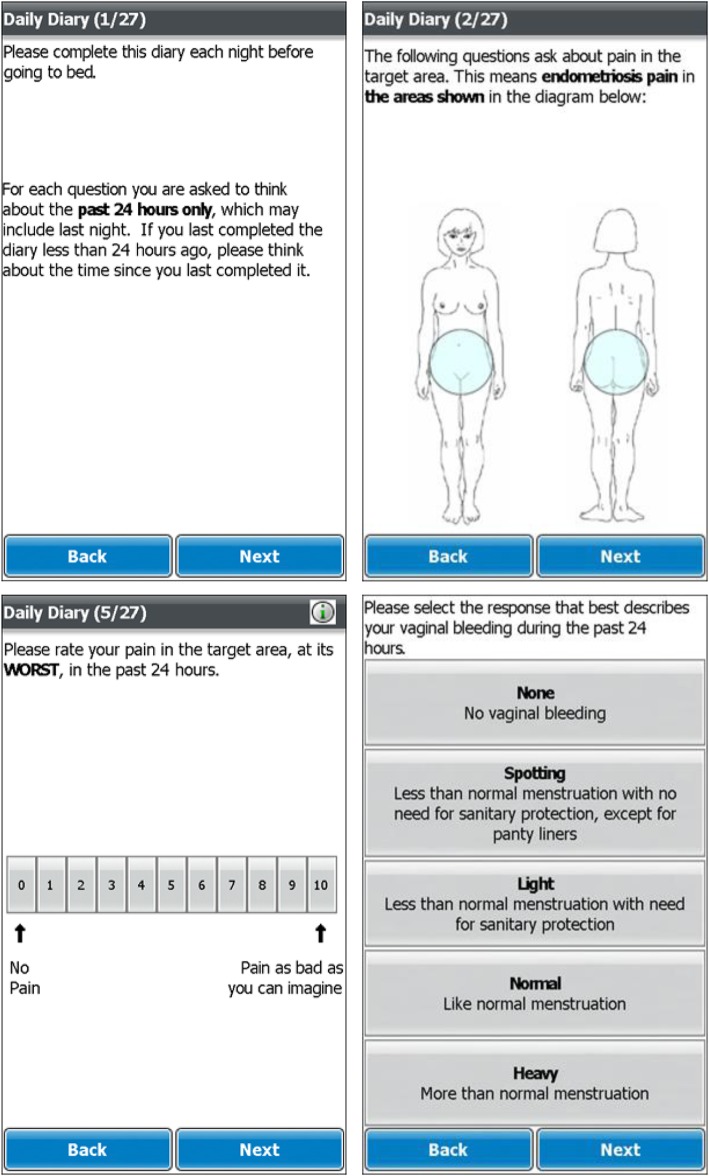
Endometriosis Symptom Diary (ESD) example US-English screenshots

The conceptual framework of the ESD and example screenshots are provided in Figs. 3 and 4, respectively.

#### EIS

The EIS was developed as a PRO measure providing a comprehensive assessment of the impact of endometriosis pain on various facets of women’s lives. In accordance with findings from the concept elicitation interviews and IMMPACT recommendations for core outcome domains to be assessed in chronic pain clinical trials [50], the primary focus of the EIS is assessment of the impact of endometriosis symptoms on women’s physical activities, emotional well-being and sexual activities.

The EIS is designed to be completed using an ePRO device, once weekly with a recall period of the past 7 days. This recall period was selected as the optimum compromise between potential recall problems and responder burden. Subsequent research has demonstrated that for assessment of the impact of endometriosis pain on physical activities, a 7-day recall period provides data that is consistent with daily administration of the same items (with a 24 h recall period) over the same period [51].

The draft EIS comprised 32 items. All items in the EIS use a 5-point verbal rating scale (‘Not at all’, ‘Slightly’, ‘Moderately’, ‘A lot’, ‘Extremely’), with an optional ‘does not apply’ option included for concepts that may not be relevant to respondents (e.g., sexual activities). This is consistent with other PRO measures investigating the impact of endometriosis / comparable conditions such as the Endometriosis Health Profile-30 (EHP-30) [52] and the Menorrhagia Impact Questionnaire [53].

**Fig. 5:**
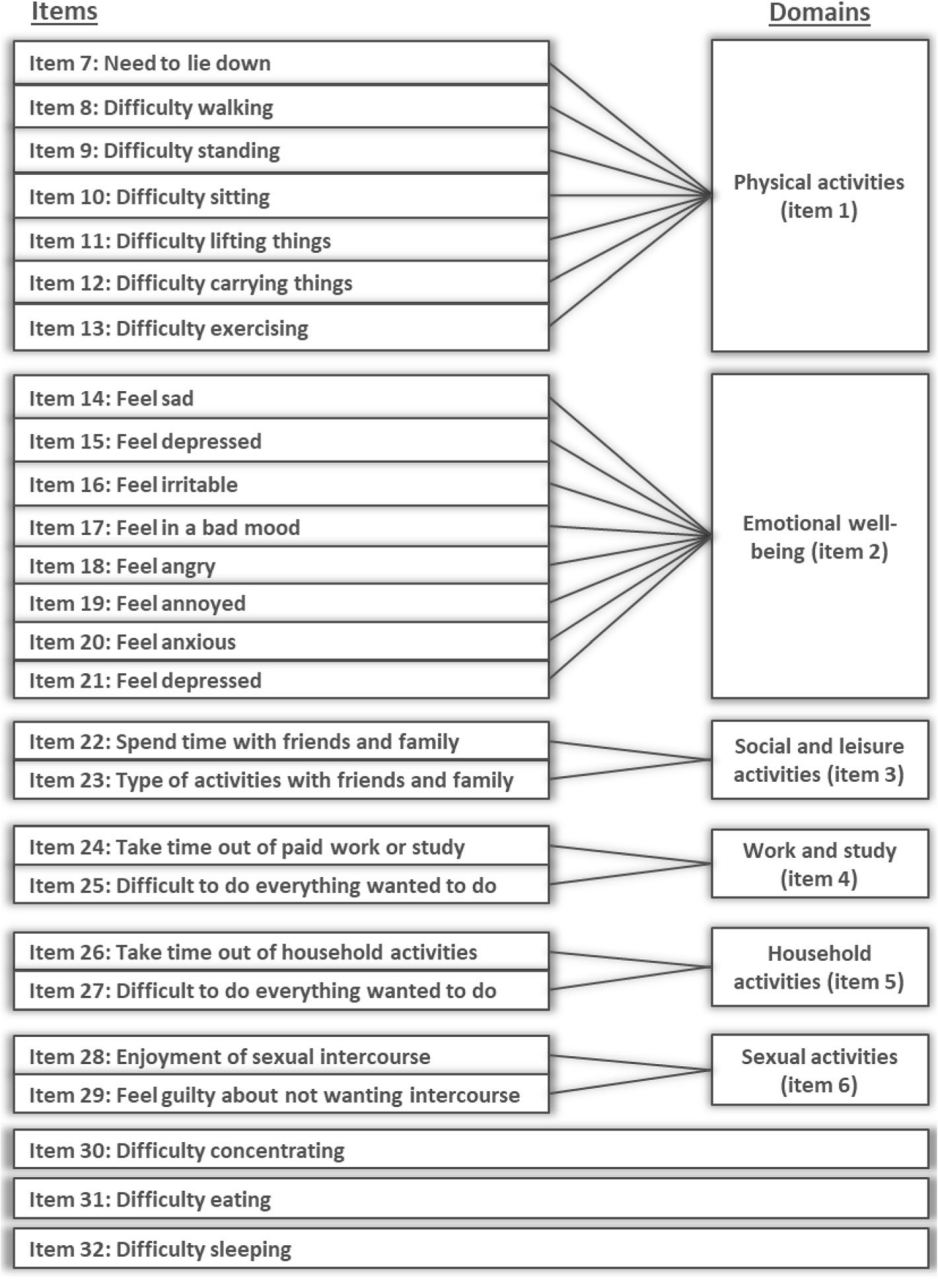
Endometriosis Impact Scale (EIS) conceptual framework

**Fig. 6:**
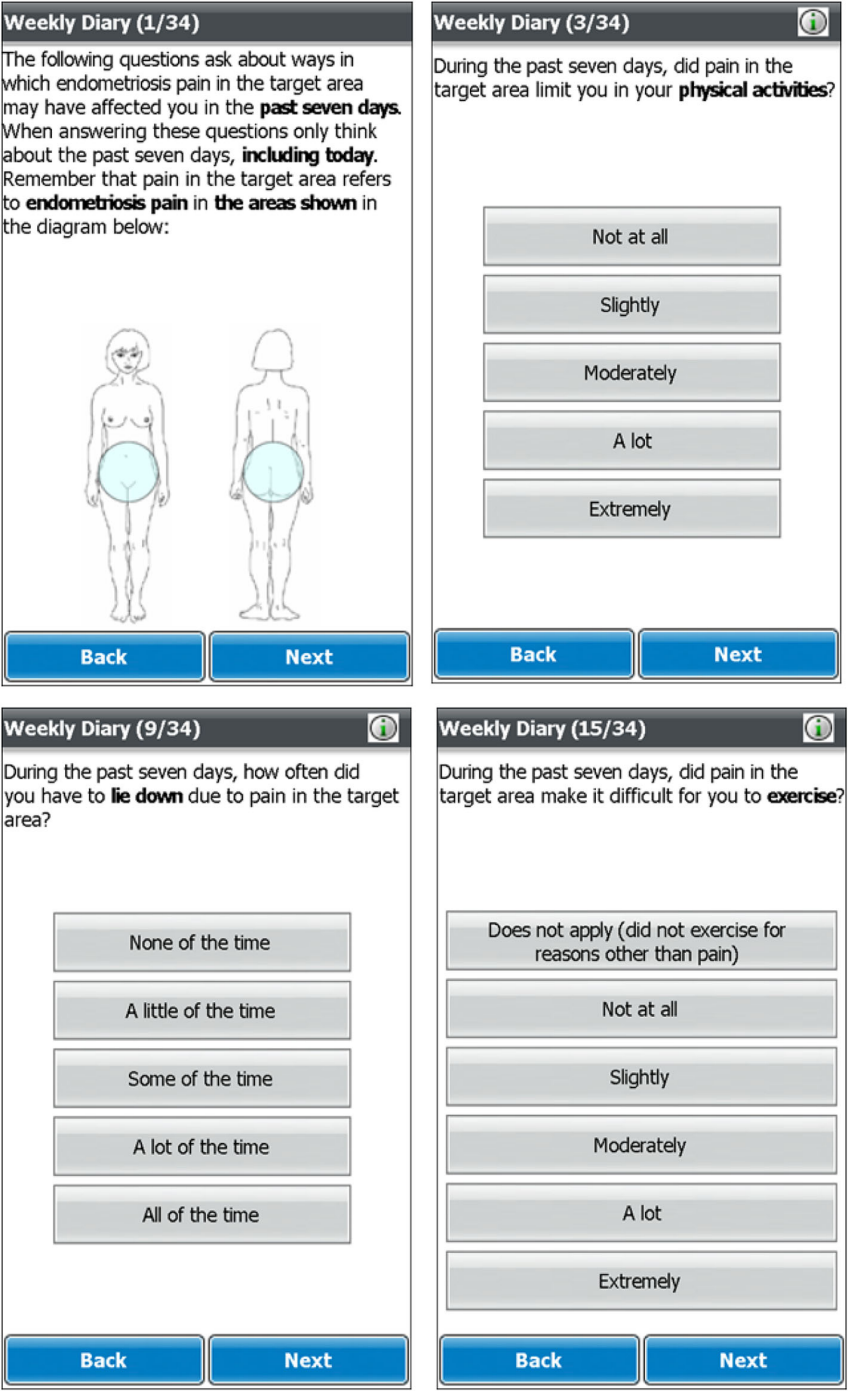
Endometriosis Impact Scale (EIS) example US-English screenshots

The conceptual framework of the EIS and example screenshots are provided in Figs. 5 and 6, respectively.

### Stage IV: cognitive interviews

Demographic and clinical characteristics of the 31 women with endometriosis who participated in the cognitive interviews are summarised in Table 1.

#### Conceptual coverage

Concepts and descriptions to emerge from open-ended discussion with participants during the cognitive interviews were in line with findings from the concept elicitation interviews. When asked during the cognitive interviews, participants indicated that the ESD and EIS captured all symptoms and impacts associated with their experience of endometriosis. There were no concepts within the ESD and EIS that were deemed to not be relevant, although some conceptual overlap between items (particularly those comprising the emotional well-being domain of the EIS) was noted.

#### Understanding and interpretation of the ESD and EIS

Feedback from participants indicated that ESD and EIS instructions, items and response options were well understood and consistently interpreted. All participants demonstrated understanding of the term ‘target area’ and all participants reported that the target area depicted on the diagram covered those areas where they experience pain related to their endometriosis. Participants were able to select responses using both the 0–10 NRS employed by the majority of ESD items and the 5-point verbal rating scale used by EIS items. Feedback from participants during the ‘think-aloud’ exercise highlighted correct use of ESD and EIS recall periods with participants thinking back to the past 24 h and past week for the ESD and EIS, respectively. Of note, no differences in understanding and comprehension were observed between US and German participants.

In light of the feedback from participants during the first round of cognitive debriefing some minor changes in the wording of ESD instructions and items were implemented and tested during round 2.

#### ePRO device usability

All participants found the ePRO devices easy to use. Of particular note, no issues regarding visual presentation, selection of responses or navigation were identified. Furthermore, good compliance, low levels of missing data and short average completion times were observed for daily completion of the ESD (2.5 min) and weekly completion of the EIS (5.45 min) during the 7–10-day completion period which allay any potential concerns regarding responder burden.

## Discussion

Establishing content validity is critical for any PRO measure. This is especially the case for any PRO measure intended to be used in clinical studies to support product labelling claims regarding treatment benefit, as evidence of other types of validity (e.g., construct validity) or reliability (e.g., reproducibility of scores) will not overcome problems with content validity [21]. The extensive evidence compiled from qualitative research among women with endometriosis and presented here serves as a critical foundation for the content validity of the ESD and EIS. Specifically, findings confirm that the ESD and EIS assess the key symptoms and impacts of relevance and importance to women with endometriosis and that both measures are understood and interpreted consistently by respondents. Exploration of linguistic and cultural differences in the way in which women with endometriosis talk about their experiences within the published literature are limited, but findings from the present study revealed the conceptualizations of women’s experiences of endometriosis to be very similar across the US, Germany and France. The simultaneous development of the ESD and EIS in the US and Europe is a key strength, with care taken to ensure the wording of instructions, items and response options are appropriate for women regardless of language spoken or literacy levels. Originally developed in US-English, French, and German, the ESD and EIS have subsequently been translated and linguistically validated for use in approximately 30 languages across Europe, North America, South America, Africa, Asia and the Middle East.

The ESD and EIS have both been employed in non-interventional (NCT01643122) and interventional research studies (NCT02203331; NCT01822080 [ESD only]). Preliminary insights from these studies have further supported the usability and feasibility of daily assessment of endometriosis symptoms and weekly assessments of endometriosis impacts using ePRO measures, with high levels of compliance and low levels of missing data observed [54]. As the next step in the development and validation of the ESD and EIS, data from these studies is to be used to evaluate item performance and to determine preliminary scoring algorithms for the ESD and EIS. The scope of these analyses will be to identify any poorly performing or redundant items and to understand the relationship between item scores to determine optimal derivation of domain or total scores. Once final item content and provisional scoring algorithms for the ESD and EIS have been established then further analyses will be conducted to evaluate the measurement properties and psychometric validity of such scores. In particular the ability of ESD and EIS scores to produce consistent scores overtime in a stable patient population (test-retest reliability), the extent to which ESD and EIS scores reflect scores for other PROs measuring similar/dissimilar concepts (concurrent validity), are able to discriminate between groups according to key indicators e.g. severity (known groups validity) and are able to detect change when the clinical status of respondents has changed (responsiveness) will be explored. Furthermore, definitions of meaningful changes in ESD and EIS scores will be explored.

There are unique challenges associated with the development, psychometric evaluation and implementation of daily diaries such as the ESD [55]. In particular, despite being favoured by instrument developers, the process by which daily diary assessments are scored and translated into meaningful and responsive endpoints has received little attention in the literature. This is particularly important in the current context where the daily assessment of pain and bleeding represents numerous options for understanding patient experiences of symptoms over time (e.g., average pain over observation period, worst pain over observation period, pain on bleeding days, pain on non-bleeding days) as well as change in these symptoms over time (absolute change in symptom severity scores vs relative change in symptom severity scores). As such, alongside research activities designed to evaluate the measurement properties of such scores, additional qualitative research is also on-going to explore patient and clinician perspectives regarding derivation of scores and definitions of clinically important differences.

PRO development can be a lengthy process and, as an area of considerable unmet need, it is not surprising that during the development and validation of the ESD and EIS, similar efforts were underway by other researchers and study sponsors to develop PRO measures for use in this area. For example, evidence regarding the development and content validity testing of another daily diary, the Endometriosis Pain Daily Diary (EPDD), has recently been published [56]. Encouragingly, concepts assessed by the EPDD and ESD are remarkably similar, supporting the content validity of both measures. However, despite these similarities there are notable differences between the ESD and EPDD. For example, a key feature of the ESD (and the EIS) is graphical depictions to aid respondents in reliably and consistency identifying endometriosis-related pain. The ESD also includes assessment of continuous and short-term pain as well as assessment of vaginal bleeding severity (rather than just the presence or absence of bleeding) which may be valuable depending on the mechanism of action for investigative products under evaluation for the treatment of endometriosis. That the ESD is complemented by the EIS is also a key strength given the absence of comprehensive PRO assessments of the impact of endometriosis on women’s lives that do not overburden patients and meet current regulatory expectations.

Finally, while the ESD and EIS have been developed to meet the guidelines and expectations for PRO measures intended to assess endpoints to evaluate the efficacy of new treatments in clinical studies, these measures may also have utility for use in clinical practice in the future. For example, many women with endometriosis experience significant delays in receiving a formal diagnosis of endometriosis (approximately 7–12 years) [57, 58]. Assessment of the core symptoms of endometriosis from the patient-perspective could facilitate earlier diagnosis of endometriosis, however there are currently no validated measures routinely used by healthcare professionals for this purpose [59]. Similarly, the ESD and EIS may have use for monitoring response to treatment in clinical practice and providing additional insights into the burden of disease beyond generic HRQoL (e.g., SF-36) and legacy PRO measures (e.g., EHP-30) commonly used for this purpose [60].

## Conclusions

The ESD and EIS are newly-developed PRO measures that have demonstrated content validity for assessment of endometriosis-associated key symptoms and impacts. Developed in accordance with the scientific best practice (including the FDA PRO Guidance), these measures are expected to have important applications for use to assess clinical trial endpoints to support regulatory label claims and for use in clinical practice to inform treatment decisions.

## Data Availability

Written transcripts of participant discussions that have been transcribed and translated verbatim are the primary source of data supporting the initial development of the ESD and EIS. While consent signed by participants indicated that quotes and excerpts may be made publicly available (i.e., via publication), consent to share entire transcripts publicly was not obtained. Therefore, it is not considered appropriate to make the data for this study publicly available.

## Abbreviations

B&B: Biberoglu and Behrman Scale
EHP-30: Endometriosis Health Profile-30
EIS: Endometriosis Impact Scale
EMA: European Medicines Agency
ePRO: Electronic patient-reported outcome
ESD: Endometriosis Symptom Diary
EU: European Union
FDA: Food and Drug Administration
HRQoL: Health-related quality of life
ISPOR: International Society for Pharmacoeconomics and Outcomes Research
NRS: Numeric rating scale
PRO: Patient-reported outcome
SF-36: 36-item Short Form Health Survey
US: United States

## Footnotes

1 Number represents unique participant ID. IDs with a prefix of 1–3 present interviews with US participants (Philadelphia, Los Angeles and New Orleans). Prefixes of 4 and 5 represent participants from Germany and France, respectively.
